# An Overview of Venous Abnormalities Related to the Development of Lesions in Multiple Sclerosis

**DOI:** 10.3389/fneur.2021.561458

**Published:** 2021-04-26

**Authors:** E. Mark Haacke, Yulin Ge, Sean K. Sethi, Sagar Buch, Paolo Zamboni

**Affiliations:** ^1^Department of Radiology, Wayne State University, Detroit, MI, United States; ^2^Department of Radiology, Center for Biomedical Imaging, NYU Grossman School of Medicine, New York, NY, United States; ^3^Vascular Diseases Center, University of Ferrara, Ferrara, Italy

**Keywords:** multiple sclerosis, susceptibility weighted imaging, MS lesion development, USPIO-enhanced magnetic resonance imaging, vascular abnormalities

## Abstract

The etiology of multiple sclerosis (MS) is currently understood to be autoimmune. However, there is a long history and growing evidence for disrupted vasculature and flow within the disease pathology. A broad review of the literature related to vascular effects in MS revealed a suggestive role for abnormal flow in the medullary vein system. Evidence for venous involvement in multiple sclerosis dates back to the early pathological work by Charcot and Bourneville, in the mid-nineteenth century. Pioneering work by Adams in the 1980s demonstrated vasculitis within the walls of veins and venules proximal to active MS lesions. And more recently, magnetic resonance imaging (MRI) has been used to show manifestations of the central vein as a precursor to the development of new MS lesions, and high-resolution MRI using Ferumoxytol has been used to reveal the microvasculature that has previously only been demonstrated in cadaver brains. Both approaches may shed new light into the structural changes occurring in MS lesions. The material covered in this review shows that multiple pathophysiological events may occur sequentially, in parallel, or in a vicious circle which include: endothelial damage, venous collagenosis and fibrin deposition, loss of vessel compliance, venous hypertension, perfusion reduction followed by ischemia, medullary vein dilation and local vascular remodeling. We come to the conclusion that a potential source of MS lesions is due to locally disrupted flow which in turn leads to remodeling of the medullary veins followed by endothelial damage with the subsequent escape of glial cells, cytokines, etc. These ultimately lead to the cascade of inflammatory and demyelinating events which ensue in the course of the disease.

## Introduction

Multiple sclerosis (MS) is usually described as an inflammatory, demyelinating, autoimmune disease (1). Conventionally, it is thought that the autoimmune processes may lead to a breakdown of the capillary and venous wall with increased perivascular inflammatory cells and other detrimental factors which attack the myelin sheath and lead to neurodegeneration (2). In this overview, we will review the literature focusing on the changes in the venous vasculature and inflammation, which are likely to be the earliest processes in the development of the disease. This review will lead us to the chicken and egg question as to whether there is venous damage that precedes the inflammatory response or inflammation that leads to vasculitis followed by an autoimmune response and then demyelination.

In general, for this review, the PubMed (www.ncbi.nlm.nih.gov/pubmed/) database was used and the search terms were (venous pathology) AND (MS OR multiple sclerosis) with a date range of 1940—present, which resulted in 900+ papers. Out of these 900+ papers, roughly 200 were reviewed in detail. This review process led us to many collateral papers that might link key pieces of information on the topics of vascular remodeling, venous collagenosis, abnormal venous flow, perfusion, endothelial dysfunction and vascular endothelial growth factors. The focus in most of these papers was on clinical studies involving human subjects, but studies on animal models were also considered if the results were directly related to the hypotheses drawn from the human subjects. The primary aim of this review was to provide an overview to non-clinical and clinical readers alike, particularly for biomedical researchers who would like study vascular involvement in MS. For an in-depth discussion of the topics covered, the readers should refer to the original publications.

These papers included some systematic reviews that provided current knowledge of the available evidence of venous vascular damage as it relates to the pathology of MS, including some very recent publications. Three comprehensive recent reviews cover the history of the vascular aspects of MS (3–5). These reviews show that there has been evidence dating back to the works of Cruveilhier, Rindfleisch [originator of the central vein observation] (6), Ribbert (7), and Charcot (8) and Bourneville and Guérard (9) in the mid-nineteenth century regarding the presence of venous abnormalities in MS. The outline of this review considers the role of: 1) inflammation and its relationship to vasculitis; 2) collagenosis and vessel narrowing; 3) ischemia; 4) infarction and abnormal venous flow; 5) abnormal bulk dural sinus flow; 6) abnormal perfusion; 7) fibrin deposition; 8) vascular abnormalities preceding vasculitis; 9) medullary vein flow; 10) central veins; 11) dilated medullary veins; 12) endothelial dysfunction; 13) medullary vein density; 14) microvascular remodeling; 15) early vascular changes as a marker for new lesions; 16) advanced microvascular magnetic resonance imaging (MRI) using ultra-small superparamagnetic iron oxides (USPIO); and 17) imaging indications of venous abnormalities. All these points help us in understanding the putative timeline of events in the pathophysiology of MS related to these venous vascular effects. Although the source of the progression of MS is currently not clear, we deduce that it may be related to abnormal flow causing the subsequent events of vasculitis, leading to a cascade of increased local blood pressure due to lumen narrowing, inflammation, extravasation of cytokines, microglial cells, peptides, etc., that promotes both demyelination and further reduction of vascular compliance, eventually leading to cell death and atrophy; or can be caused by the endothelial dysfunction due to early increase in pro-inflammatory cytokines leading to vasodilation and vascular remodeling that is typical to MS, followed by chronic hypoperfusion in the later degenerative stage of the lesion. This comprehensive overview of vascular effects in MS should open the door to study venous abnormalities and abnormal venous flow and their temporal relationship to the development of MS lesions using a variety of *in vivo* imaging methods.

The early pathological understanding evidenced throughout this work suggests a major role for the veins in MS, however, the temporal evolution still remains unclear: “Does poor flow generate the endothelial response and vessel wall inflammation or does the abnormal flow lead to ischemia and vessel wall breakdown including inflammation and vasculitis?.” In both cases, there will be an extravasation of auto-immune invoking products that follow these processes. As we progress through the different forms of evidence, the role of abnormal flow will occur again and again as a potential source initiating the inflammatory response. After this, the usual cascade of damaging events to the tissue and demyelination will occur.

### Inflammation and Vascular Damage

Early cadaver brain studies of MS patients showed that the veins and venules in or at a distance from active lesions frequently exhibited an inflammatory lymphocytic reaction essentially located only in the vessel wall (10). When confined to the venous wall, these inflammatory changes can be regarded as a form of local venous vasculitis or cerebral venulitis. In this work, they showed that the cerebral venular wall in multiple sclerosis is the site of lymphocytic infiltration that may, at first, particularly in grossly normal white matter, be confined to the vessel wall alone (10). As the inflammatory process proceeds, the cellular infiltrate appears to spread to the perivascular space and even into plaque tissue. This may occur without any obvious surrounding demyelination (11). Venous walls then undergo focal intimal hyperplasia, intimal organization and collagenous thickening. Such features suggest a mild expression of subacute or chronic endovenulitis of the cerebral veins. Chronic inflammation can also lead to hemorrhage, increased permeability of the vessel wall and vasculitis. In the absence of other pathology, it is reasonable to assume that the scarring results from early inflammatory changes. The pathogenic mechanisms that cause the cerebral venulitis in multiple sclerosis could be the deposition or penetration into the venous wall of some circulating factor from the blood, a specific lesion of the venous wall or the release of a substance from the damaged brain, such as a lysosomal enzyme or thromboplastin (10). Adams further showed that in some active acute cases of multiple sclerosis the venous walls themselves are heavily infiltrated with inflammatory cells, but without infiltration of the adventitia and perivascular tissues (12). Chronic plaques often contain venous walls thickened by collagen, whereas active inflamed lesions exhibit a more fibrinous exudate (10). Although these processes are non-specific, they do indicate that the venous wall is implicated in the inflammatory process, and is thereby damaged and thickened, partly analogous to the thickening in endarteritis obliterans in arteries passing through foci of chronic inflammation. Nevertheless, it could be held that the venous wall is merely acting as a conduit for inflammatory cells but, if so, it should return to normality after subsidence of inflammation as, in fact, it does in MS. These works show that the venous wall is affected during the process of developing MS. Some have gone so far as to hypothesize that one of the fundamental components of MS is a form of auto-immune vasculopathy (5).

### Venous Collagenosis

Another major effect seen in the vasculature of MS and other diseases is collagenosis, a process that affects connective tissue and is often associated with fibrinoid necrosis or vasculitis. In their work on stroke, Black et al. comment on the role of arteriolar tortuosity, reduced vessel density and occlusive venous collagenosis (gradual thickening of the vessel wall from collagen build up) which causes venous insufficiency and vasogenic edema (13). The key markers and evidence for this are activated microglia, oligodendroglial apoptosis, clasmatodendritic astrocytosis, and upregulated hypoxia-markers. Gao et al. proposed that venous collagenosis dilates the veins and causes venous insufficiency with consequent vessel leakage, i.e., vasogenic edema (14). Moody noted that the blood-brain barrier (BBB) is abnormally breached in conditions such as hypertension, intra-arterial injections of hyperosmolar solutions, exposure to inflammatory mediators (cytokines), activation of leukocytes, hypercapnia, and ischemia (15). With serious injury to the BBB, large amounts of plasma proteins can escape into the brain tissue, resulting in vasogenic edema. Brown et al. show that collagenosis of the veins could prevent exchange between the surrounding CSF and the venous blood that may act as a drainage for waste products of the brain (16). Further, stiff arteries may not provide the needed pulsatility to drive the CSF and again reduce macromolecular transport to the CSF from the brain and eventually to the venous blood (17). These effects may affect the function of what is referred to today as the glymphatic system (18).

Moody et al. proposed that periventricular venous collagenosis (PVC) could lead to venous wall thickening of the periventricular and subependymal veins, luminal narrowing and eventually vessel occlusion (19). They also associated this with leukoaraiosis in elderly individuals when there was no evidence of small arterial disease. They believed this lack of flow could lead to: reduced perfusion pressure, increase in associated venous pressure, chronic cerebral edema, disordered venous flow, BBB breakdown, ischemia infarct and hemorrhage (19, 20). The development of leukoaraiosis may be slow when PVC is present because of the presence of collateral flow and alternate pathways in these areas (although such pathways are not endless). They also note that the obstruction of flow might interfere with the “paravascular” channels and the exchange of extracellular fluid to the systemic circulation. Further, the collagenic venous thickening is likely to impede resorption of the CSF and extracellular fluid. They note that the thicker wall might prevent rupture and venous collapse when there is increased ventricular cerebrospinal fluid pressure or vigorous pulsations. This can lead to increases in transmural pressure preventing the usual flow of metabolic wastes into the venous system (21). Houck et al. focus on the role of medullary venule collagenosis and associated vasogenic edema in white matter hyperintensities (WMH) and Alzheimer's disease (AD) (22). They studied 682 older adults without dementia and found an increase in diameter of the internal cerebral veins and basal veins of Rosenthal were associated with greater total WMH volume in different regions of the brain. Keith et al. showed in a cadaver brain study of dementia and healthy controls (HCs) that collagenosis of venules best predicted WMH volumes (20). They note clearing of interstitial solutes such as beta-amyloid occurs via the glymphatic system associated with the perivascular spaces. Therefore, if venous dilation affects paravascular spaces, the glymphatic system could be disrupted.

Despite the fact that these papers are referring to stroke, hypertension and dementia, the findings for the early role of the medullary venous system are clear, their flow and perhaps function have been compromised.

### The Role of Venous Ischemia

Yan et al. discussed the role of WMH in general in other diseases excluding MS but we can learn from this data too; there may well be some hints of the mechanisms including dilated veins (23). Increased levels of WMH are associated with an increased risk for dementia and stroke. There is often reduced blood flow in these regions with BBB damage (24, 25). Cerebral venous collagenosis (VC) may cause “venous ischemia by increasing vascular resistance and compromising interstitial fluid circulation, with consequent vessel leakage, i.e., vasogenic edema, and lead to non-necrotic hyperintensities on MRI (13, 19).” The resulting reduced venous outflow and venous hypertension may lead to dilation of deep medullary veins (DMV) (26). WMH could be induced by stenosis or occlusion of deep cerebral veins (26). In addition, retrograde venous hypertension could lead to decreased cerebral blood flow (CBF), venous ischemia and hypoxia (27). Willinsky et al. and Van Den Berg et al. also note that there can be partially reversible WMHs in patients with arteriovenous malformations (28, 29) and that venous ischemia was more likely to be a cause of WMHs.

Keith et al. (20) used cadaver brains to stain for venous wall thickening and occlusion caused by collagenosis. Although they found higher venous collagenosis for smaller veins 150 μm and less, VC in larger veins (>200 μm) correlated with higher WMH scores. They also found VC was frequent in patients with periventricular infarcts identified on imaging in both AD and non-AD patients. The etiology of WMH remains unclear although there is an association with cerebrovascular disease and hypertension potentially leading to ischemia. Their previous work also suggested that venous insufficiency and vasogenic edema may be factors in the development of WMH (13) and may be present in cerebral autosomal dominant arteriopathy with subcortical infarcts and leukoencephalopathy (CADASIL) as well (30). Small vein collagenosis and white matter pallor was more prevalent in the presence of arteriosclerosis. *They assume white matter pallor represents myelin loss*. In summary, periventricular venous infarction accounts for 22% of ischemic stroke in adults and 75% of subcortical lesions in perinatal stroke.

These works continue to develop the concept that the veins can play a major role in ischemia and lead to white matter hyperintensities.

### Cerebral Venous Infarction

Schaller and Graf show that when cerebrovascular occlusion (CVO) is present, the local pressure increases leading to: a dilated venous and capillary bed, the development of interstitial edema, increased CSF production, decreased CSF absorption and rupture of venous structures (hematoma) (26). If promptly diagnosed, CVO is reversible and this can avoid venous infarction. They note the following: (a) an increase in venous and capillary pressure leads to diapedesis of erythrocytes by BBB disruption; (b) dilatation of venules can occur with no arterial effects; (c) reduced CBF leads to an acute energy drop, dysfunction of the membrane-bound Na + –K + –ATPase pump, intracellular entry of water, cytotoxic edema and cell lysis. These phenomena can be considered as a disruption of the shift of interstitial fluid from the capillary bed toward the ventricle. The key is that despite the immediate metabolically disturbed state after CVO, the effects may be reversible, which is certainly true in MS as long as adequate perfusion has been maintained during the stressed conditions. This is likely made possible by venous anastomosis acting as the collateral pathways. The other thing to remember is that venous flow can change direction since there are no valves in the veins. Therefore, subsequent dilation of the veins and recruitment of (or remodeling of) neighboring outflow territories may temporarily compensate for changes in pressure. If pressure increases beyond some limit, this could cause BBB disruption and alter the fluid exchange between cerebral intra- and extravascular compartments, causing cytotoxic and vasogenic edema. This protective nature of alternate venous pathways has been shown in rat studies of venous occlusion (31). Increased pressure and tissue swelling can also cause collapse of the capillaries.

This work shows that under abnormal flow conditions, veins can be damaged, dilate and restrict blood flow disrupting the BBB and fluid exchange in the glymphatic system.

### Dural Sinus Flow Effects, Abnormal CSF Flow, and Increases in Venous Pressure

Several authors have shown a combination of intraluminal obstacles and/or external compression, hampering the venous outflow at the extracranial level, particularly in the internal jugular veins (IJVs) (32–37). According to fluid dynamic simulations using a mathematical model based on human vascular structure, these dural sinus flow abnormalities can lead to an increased pressure in intracranial veins (38, 39). They show that when both IJVs are obstructed, there can be a rise in pressure of up to 13 mm Hg (38). This might explain the dilation of the cerebral venules and medullary veins assessed by Gaitán et al. (40), in consequence of the increased transmural pressure at that level. It has been shown in a rat model that an embolus in the external jugular can produce large venous pressure changes in the superior sagittal sinus and subsequently a loss of CBF and accumulation of nicotinamide adenine dinucleotide (NADH) in cortical structures indicating ischemic damage (31).

CSF is ultrafiltrated from the high-pressure capillary bed of the choroid plexus into the subarachnoid space. From the subarachnoid space, CSF is driven into the Virchow-Robin spaces by a combination of arterial pulsatility, respiration, and pressure gradients. When blood reaches the low pressure system of the cerebral venules, water is reabsorbed into the venous system from the interstitial fluid and CSF (41). CSF absorption is linked with a gradient of pressure between the brain parenchyma and the venules, constituting the so called G-lymphatic system (42–44). When IJV obstruction occurs, the increased venous pressure is propagated to the intracranial sinuses and venules. The effects of higher pressure in the cerebral venous system are, therefore, significant for any fluid exchange, because either the G-lymphatic fluid absorption or the CSF passage into the dural sinuses is based on a favorable pressure gradient (43, 44). In case of extracranial venous obstruction, the increase in pressure at the venular level can affect the absorption of peptides, triggering the inflammatory process (44), and affect the passage of macromolecules (17). One also observes in MS that the ventricles are enlarged relative to controls as are the perivascular spaces close to venules (45). Magnano et al. showed that CIS patients had net reduced CSF flow and that their conversion to clinically definite MS in the following year was related to this decreased CSF flow (46). The perivenous spaces in the brain parenchyma are increasingly recognized for their role in leukocyte trafficking as well as for their potential to modulate immune responses and, therefore, might be considered biomarker of inflammation. Finally, increases of macromolecular concentrations in the perivascular spaces caused by impaired transport across the venous wall could lead to edema (17).

These papers demonstrate that changes in flow whether microscopic or macroscopic that affect the venous system upstream can cause pressure changes leading to further exacerbations of abnormal flow.

### Changes in Perfusion of MS Lesions and Normal Appearing White Matter (NAWM)

A number of studies have shown that there is a general reduction of perfusion in chronic lesions in MS and in NAWM as well (47–52). One paper notes a reduced CBF by about 25% and increased mean transit time (MTT) of 3 to 4 s in MS lesions (52). The trend of lower CBF and cerebral blood volume (CBV) in NAWM became worse in primary progressive MS (PP-MS) and Expanded Disability Status Scale (EDSS) was significantly correlated with the periventricular CBF and CBV as well as the frontal CBV (53). Reduced CBV was found to correlate with working and secondary verbal memory for clinically isolated syndrome (CIS) patients (54). Another paper showed reduced CBF in NAWM and decreased NAA/Cr in the centrum semiovale and increased PCr/β-ATP (51). Law et al. has also shown a decreased cerebral blood flow and a prolonged mean transit time in periventricular regions of NAWM (55). Dynamic susceptibility contrast perfusion may also be used to view microvascular changes in non-enhancing lesions (56). These flow reductions in lesions and NAWM may lead to a state of hypoxia in the tissue. Generally, worse flow is associated with progression of the disease and more severe physical disabilities (57).

On the other hand, in the acute stage, there is evidence of increased CBV (58), CBF (47, 50, 59), and reduced MTT, the latter being associated with higher disease severity and with the presence of disease 1 year later in newly diagnosed MS patients (60). Another paper compared high-inflammatory and low-inflammatory patients and found that the former had significantly higher CBV and CBF values (59). Interestingly, CIS patients have also been shown to have increased CBV (61, 62). Ge et al. also show that lesions with low CBF and low CBV (basically chronic lesions) do not show enhancing lesions with gadolinium (56). An excellent review of all the literature related to MRI perfusion imaging is given in Laganà et al. (57) and on the role of chronic inflammation and imaging inflammation is given by Matthews (63).

One of the more interesting findings are the changes in blood flow and blood volume preceding the formation of acute lesions (45, 59). These effects appear to remain for several weeks even after BBB breakdown has ceased. The increase in CBF and CBV suggests a possible dramatic change in the local vasculature preceding the inflammatory stage.

The take home message from this section is that reduced perfusion can lead to loss of brain function even in CIS. Especially suggestive is the finding that blood flow changes can precede lesion formation. We propose that the odd increases in blood flow may be caused by vascular remodeling and the subsequent increase in local venous blood volume.

### Fibrin, Flow Disruption, and Ischemia

Wakefield et al. studied the role of vascular injury in MS (64). Damage to the veins causes vascular endothelial cell activation, vasculitis, vascular occlusion including class II antigen expression and fibrin deposition (usually a sign of active lesions) possibly in that order making the antigen expression an early event in the evolution of vascular injury. These changes can occur prior to cerebral parenchymal reaction and demyelination and suggest that ischemia could be an early component in the evolution of multiple sclerosis. They also note that these vascular effects were present in many thin walled vessels, including both veins and capillaries, while other vessels were occluded by reticulated fibrin thrombus. When fibrin was present, the walls of these vessels appeared widened by inflammatory infiltrate and edema. For more advanced cases, reactive astroglial cells were present around the damaged vessels. In some cases, groups of vessels were apparently obliterated and the vascular origin of the associated lesions was confirmed by collagen type IV immunostaining. Fibrin is also associated with vessel wall infiltrated by inflammatory cells, and may progress to occlusive venous thrombosis (hemorrhage was frequently seen in relation to these damaged vessels). In some experimental allergic encephalomyelitis models, fibrin formation appears to be a prerequisite for the development of clinical disease where they show disease activity began only with the appearance of fibrin in this model (65).

So, what role does focal cerebral ischemia play in the pathological and clinical features of multiple sclerosis? The preservation of axon cylinders is a recognized feature of hypoxia and is seen in leukoencephalopathy (66). Some evidence for ischemia exists from MRI where it has been shown that there is an increase in lactate (67). They note that shunting of blood from poorly perfused lesions could produce an acute symptomatic deterioration and in turn the restoration of blood flow could produce a rapid clinical improvement. This could explain the coming and going of MS lesions over time and only those lesions with chronically dysfunctional flow may become permanent. In conclusion, they propose that “focal endothelial cell activation which progresses to occlusive vascular inflammation is a precursor of both cellular infiltration of vessels and demyelination.” And they close with this comment: “Advanced vascular injury is associated with infiltration of the vessel wall by inflammatory cells and reactive changes in the neuropil. We propose that activation of the cerebral endothelium is a primary event in multiple sclerosis; that induction of procoagulant activity in endothelial cells is a feature of acute multiple sclerosis; and that demyelination may have an ischemic basis in this disease.”

The two papers by Wakefield and Ginsberg begin to strengthen this picture of ischemia, abnormal flow, fibrin deposition, and the shunting of blood from poorly perfused regions leading to acute symptomatic degeneration. The comment that a return of normal flow could lead to resolution of the inflammation and disappearance of the lesion is reminiscent of what is seen in relapsing remitting MS.

### Evidence That Vascular Abnormalities Precede Vasculitis and Inflammation

It is well-known that patients with retinal vasculitis can go on and develop MS. Retinal vasculitis has four main abnormal immunological findings: 1) macular edema; 2) periphlebitis and perivascular sheathing; 3) capillary closure and/or leakage; and 4) venous occlusion and new vessel formation (68). In periphlebitis retinae there is sheathing and hemorrhage in veins in the retina (69). Also, patients with retinal vascular abnormalities in optic neuritis have a high probability to develop MS (70) and the overall risk of being diagnosed as clinically definite is 28%. These authors note that both the brain and the retina have continuous endothelial tight junctions which are permeable to a variety of molecules (70): “*We therefore suggest that the sheathing of retinal vessels that we observed opthalmoscopically is the visible clinical sign of the perivascular lymphocytic infiltration and accompanying edema which characterizes the lesions of MS*.” and more interestingly they comment: “*The occurrence of perivenular abnormalities in a region free of myelin and oligodendrocytes provides evidence that the vascular changes in MS can occur independently of contiguous demyelination and may be the primary event in the formation of a new lesion*.”

This long-standing evidence of the relationship of poor flow with retinal vasculitis is indicative that poor flow can and does appear in some cases as a precursor to inflammation.

### Medullary Veins

The medullary veins of the brain play a key role in draining the blood from white matter. A number of diseases are associated with the medullary veins including hemorrhagic disorders, inflammatory changes that spread along the veins, and neoplasms within the veins (71). The first two may be related to metabolic changes associated with venous wall damage and are implicated in MS. It is well-known that there are pathological changes of veins in plaques that include: lymphocyte cuffs, intramural fibrinoid deposition, collagenized vessel walls, and perivenous iron deposition (12). On the arterial side, Nonaka et al. note that the presence of anastomoses among the terminal branches of the deep white matter protects against ischemic infarction (72). This type of anastomosis is likely present in the DMV as well. Okudera et al. studied the zones of convergence of the medullary veins (73). There were four zones of convergence: the superficial, candelabra, palmate and subependymal. Several zones are related to venous crossings of the fibers of the corona radiata and optic radiations. The arcuate veins may link to the cortical veins and these can be called sub-cortical veins. The deep medullary and superficial medullary veins can be connected by anastomotic veins. The longitudinal caudate vein of Schlesinger is also referred to as an ependymal vein when running beside the lateral ventricles (74). He notes that there are medullary veins around the fourth ventricle too. The superficial and deep medullary vein territories represent the watershed zone between the cortex (pial) and deep white matter (ependymal drainage). When the balance of drainage is disrupted between these two systems, the veins now responsible for the larger than normal flow will dilate. Finally, Willinsky noted that venous congestion may lead to rerouting into dilated transosseous venous channels or retrograde flow (28).

The structure of the medullary vein system could well explain the location of both the periventricular and zone 2 lesions by the candelabra medullary veins as well as the presence of transmedullary veins related to the gray matter. The medullary veins of course pervade all the tissue and a breakdown of the drainage system could have drastic consequences despite the fact that the venous system offers multiple anastomotic connections.

### Central Vein Sign (CVS)

**Figure 1:**
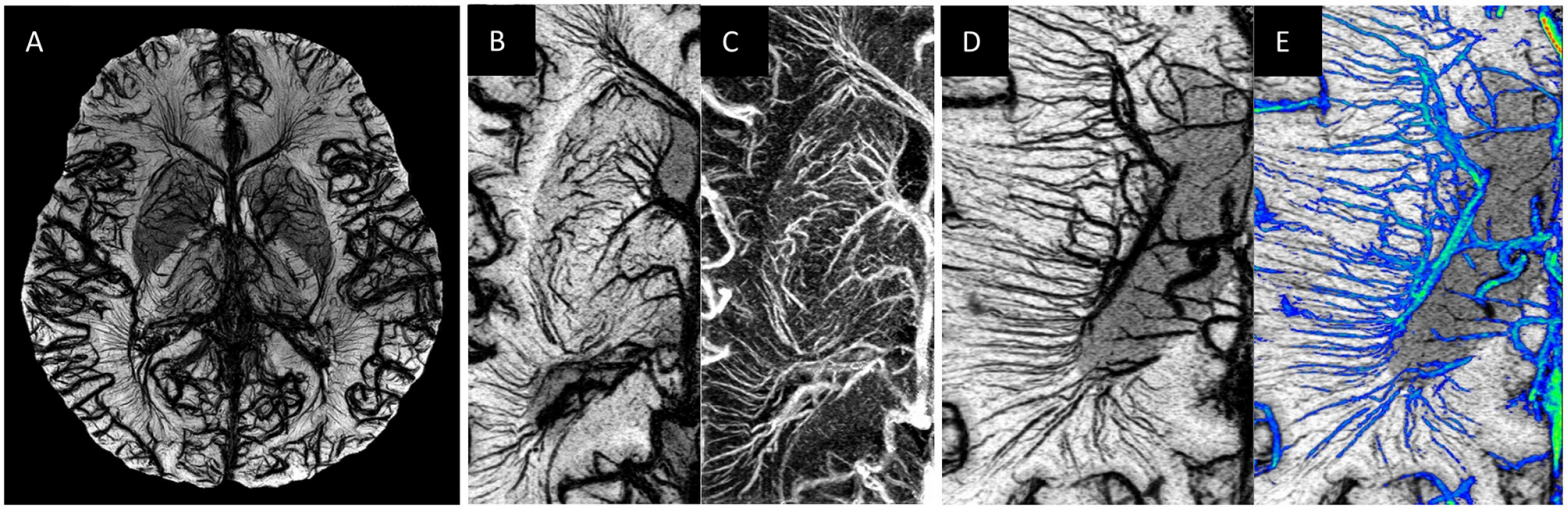
Examples of MICRO imaging: **(A)** whole brain; **(B,C)** locally in the basal ganglia (image **B** is the SWI and image **C** image is the susceptibility map) and **(D,E)** the medullary veins. For part **(D)** the Frangi vesselness mask (78) was used to separate the veins (image **E**, shown in blue) from the background tissue from which one can calculate a medullary vein density (MVD).

**Figure 2:**
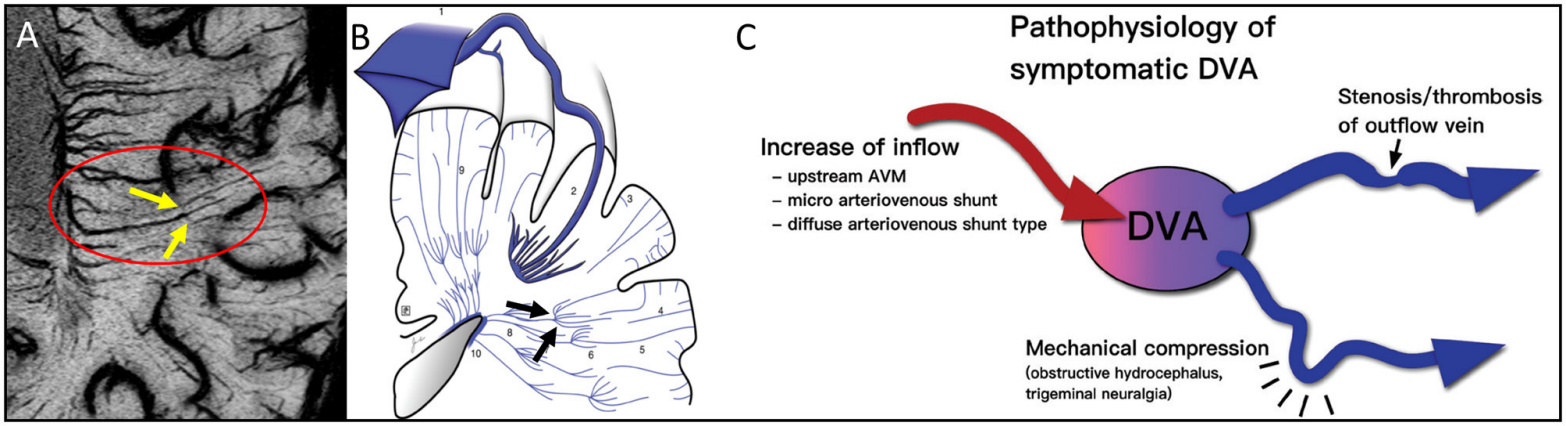
Note the clear example of the pitchfork (candelabra) seen with MICRO imaging (**A**, yellow arrows within red oval) that matches the sketch (79) from the cadaver brain work of Okudera (73) (black arrows in **B**). The idea of remodeling of the venous vasculature and recruiting anastomoses between medullary veins is not new. It is thought that these can occur for reasons of blocked flow and subsequent increases of inflow initially. This concept fits the results illustrated by the MICRO imaging data shown in Figure 3. The atypical DVA can have significant effects on the local flow and multiple clinical consequences **(C)**. Illustration taken from Aoki and Srivatanakul (79). This diagram shows that there can be increases in inflow, decreases in venous flow and mechanical compression as part of the pathophysiology of DVAs. **(B, C)** reproduced/adapted from Neurologia medico-chirurgica (NMC) under CC-BY license.

**Figure 3:**
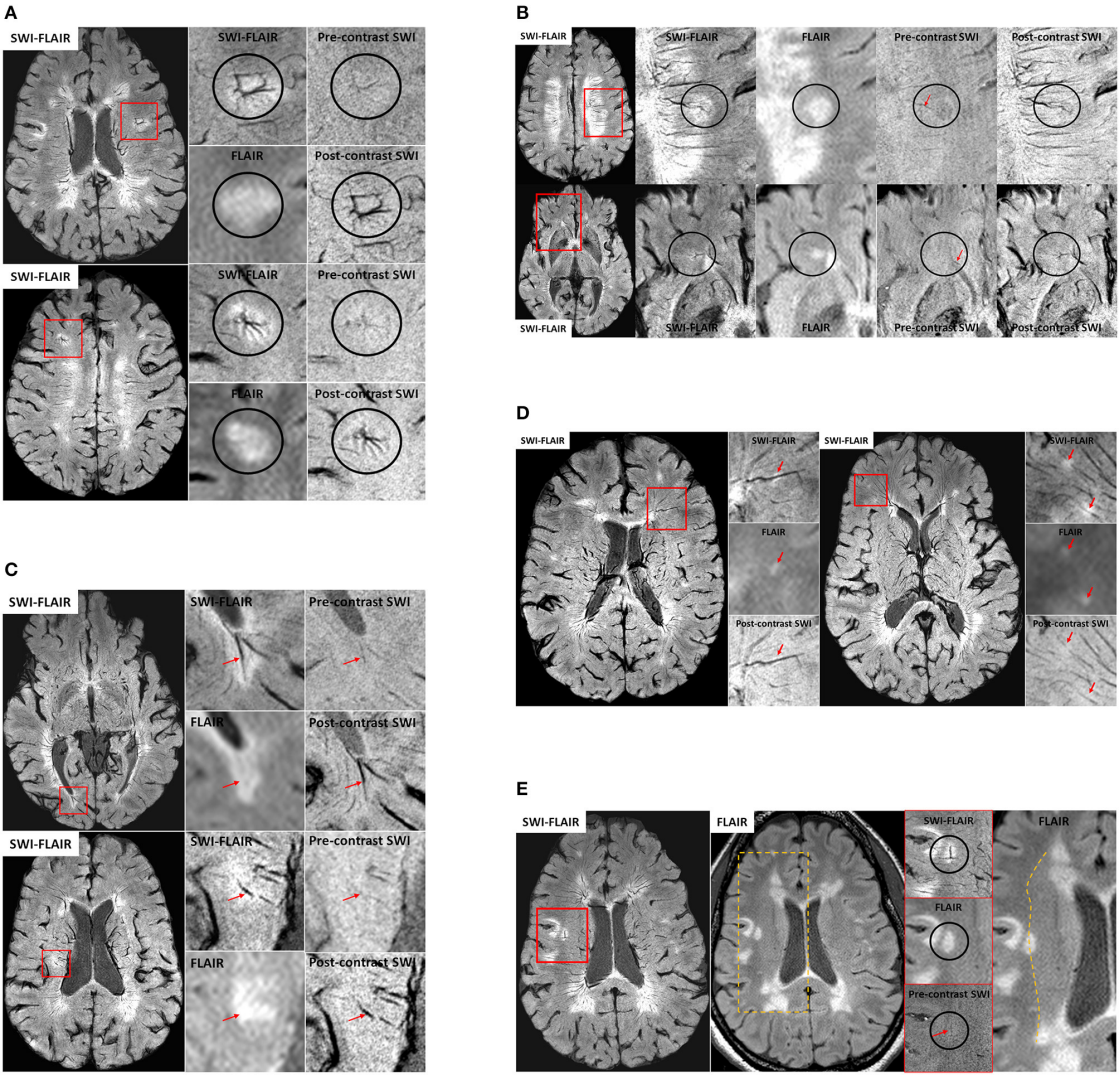
**(A)** Examples of venous angioma like abnormalities in MS lesions from two patients. The small vessels joining the lesion-centric junction are not visible in conventional imaging and hence these DVAs have gone unrecognized in the past. Only the primary draining veins were slightly visible on the pre-contrast SWI data, confirming their venous origin. **(B)** Two different examples of micro-angiomas in MS lesions. Unlike the cases for larger angiomas, the pre-contrast SWI does not show any hints of these smaller vessels seen on the post-contrast SWI or SWI-FLAIR data, except for the primary vein (red arrows) that is draining the lesion-centric spoke-like small vessels. **(C)** Two different veins exhibiting dilation with the MS plaques. SWI-FLAIR data clearly shows the abrupt change in the vein diameter within the lesion. If these are active lesions, the inflammation may continue to develop around these vessels over time. In these cases, there may not be any further gadolinium enhancement as the lesion grows due to reduction of the vessel size or the complete blockage due to collagenosis. **(D)** Small ovoid WMHs (red arrows) located along the vessel wall of the small vessels as confirmed on SWI-FLAIR, FLAIR and post-contrast SWI data. **(E)** An example case showing a potential anastomotic vein. Once again, the pre-contrast SWI data was used to confirm the venous origin (red arrow) of this lesion-centric anomaly. The medial-to-lateral change in WM intensity on the FLAIR data (dotted line on the right-most image) is suggestive of the presence of the corona radiata layer in the WM and note that the anastomotic vein is abutting at this junction.

As mentioned earlier, Rindfleisch had already noted the presence of a central vein in the mid-1800s (6). It was not until mid-1900 that this became more evident in Dow and Berglund's work (75). A major recent focus has been on the presence of a central vein in MS lesions especially in Dawson fingers and sometimes in smaller lesions as well (76, 77). This sign has been used to differentiate other white matter diseases that are often part of the differential diagnosis from MS, sometimes quite successfully (76). The presence of abnormal veins is a possible explanation of the widely held hypothesis that the formation of an MS lesion depends on the entry of inflammatory cells from the systemic circulation into the brain parenchyma possibly from a disrupted endothelium of the veins. However, not all lesions show major veins, although with the help of the Microvascular *in-vivo* Contrast Revealed Origins (MICRO) imaging approach, where a USPIO agent, Ferumoxytol, is administered and high-resolution susceptibility weighted imaging (SWI) images are acquired, we can visualize any vascularity within the MS lesions (Figures 1–**3**). One immediate question is: “Are there, in fact, veins present, but they just can't be seen?” Some evidence for this exists. One case study has shown that a central vein became visible only after the use of a Gadolinium contrast agent. One explanation for not always seeing a CVS is that reduced metabolic function of adjacent tissue leads to a loss of visibility of the medullary vein. Several authors have suggested the use of contrast to further enhance the visibility of the veins (77, 80). This can be understood as a T2*-T1 coupling for improved signal loss in SWI (81).

An early study by Broman used trypan blue staining of the MS plaques and found only veins were stained. The staining correlated with the degree of demyelination and in some cases an obvious correlation between the course and branches of the veins and the shape and extension of the plaques. He found that there were central veins in every plaque (82) and that there was some degree of correlation with blue staining and demyelination. Fog (83) stated that MS lesions typically were developed around small veins. This work also noted that 2/3 of plaques evaluated followed the veins along a classic Dawson finger profile. More interestingly, the course and size of the veins determined the shape, course and dimension of the plaques. Further evidence of the vascular mechanical effect comes from Allen's observations, who noticed the wide vascular beds around veins and the central widening of the venous tree that testifies an intermittent increase in cerebral pressure (84). This is similar to Schelling's findings (85).

In an effort to establish a standard radiological definition of the CVS to improve diagnosis of MS, Tallantyre et al. introduced the “40% rule” which assesses the number of MS lesions with CVS as a fraction of the total number of lesions. A 40% cut-off threshold was able to discriminate MS from healthy subjects (86). Sati et al. used the 40% CVS rule to separate MS lesions from other types of lesions with a small number of cases (87). Mistry et al. found 45% CVS was a good separator for MS vs. other diseases such as microangiopathy even at 3T (88). Samaraweera et al. used a high resolution segmented echo planar imaging (SEPI) sequence with echo train length (ETL) = 15 and with 0.55 mm isotropic resolution in 4 min 14 s (89). In this method, the long echoes and high CSF signal help visualize the lesions better. They also showed a much smaller fraction of CVS with patients with small vessel disease (SVD). We can use an iron-based contrast agent to enhance the visibility of CVS with a practical scanning time, or a longer TR, longer TE and lower flip angle non-invasively to try to enhance this contrast [this is particularly true for 7T (90)]. Maggi et al. studied the prevalence of CVS in MS vs. systemic autoimmune diseases and primary angiitis of the cerebral nervous system (91). Using a threshold of 50% for the perivenular lesions, all other vasculitis like diseases were ruled out. Behcet's disease had the highest perivenular lesion load of all vasculitis diseases. All of these studies have very strong predictive value in terms of differentiating MS from other diseases and further implicate the venous role in MS.

Clearly, the medullary veins are a major presence in MS lesions. However, this could be even more evident if much higher resolution imaging could be used where more “micro” level venous abnormalities could be seen. This is in fact demonstrated later in this paper under section Imaging the Microvasculature Using a USPIO Contrast Agent.

### Evidence of Dilated Veins

The behavior of the medullary veins is complicated. For example, in stroke and restricted retrograde venous hypertension, DMV could dilate or appear dilated in SWI due to the increased levels of deoxyhemoglobin. Actually physical increases are also possible if there is a source of stenosis downstream that could lead to mechanical effects such as pulsating blood and enlargement of the perivenous spaces because of this (85). These flow disturbances could lead to deleterious effects on the vessel wall allowing lymphocytes etc. to escape. Eventually, upon tissue death, the veins may also atrophy or become so constricted due to collagenosis that the effective blood volume is decreased in chronic lesions (58). Yan et al. used SWI to evaluate the volume of DMVs in patients with WMHs and HCs (23). The volumes of DMV on SWI may make it possible to monitor the severity and progression of disease associated with WMHs and to evaluate response to therapy. This venulopathy causes dilatation of upstream venule beds. Further, they found a correlation of DMV volumes with the number of lacunes as did another more recent study by Zhang et al. (92). This apparent increase in DMV could be caused by increased cerebral metabolism or reduced flow. It has been shown in subjects with large WMH volumes that there is reduced CMRO_2_ (93, 94). Black et al. proposed that venous collagenosis could dilate the veins and damage the myelin and axons with the consequent leakage of potentially toxic substances (13). It has been shown that the DMV were not only diminished in density (95), but also shortened and sometimes dilated and for early patients can appear longer (96). Adams noted that chronic plaques showed thick-walled vessels and little lymphocytic infiltration (12). Usually, less blood flow would lead to an increase in the visibility of veins in SWI under normal tissue functioning. However, if the tissue is not functioning at capacity, then there will be less deoxyhemoglobin and there will be a loss of visibility in SWI.

Gaitán et al. showed that the veins found inside MS lesions were smaller than the veins outside MS lesions (40). They also found that the veins located around and outside lesions in people with MS were larger than the veins in people without MS. They proposed that compression within the active lesions by the perivascular cuffing or hardening of the vascular wall (like necrosis) causes reduced vascular compliance in chronic lesions. They also suggest these enlarged extralesional veins could be due to: ex vacuo dilatation due to overall brain volume loss or an apparent increase from T2* blooming artifact caused by an increase in the amount of deoxyhemoglobin in the blood. Assuming that arterial blood in MS is normally oxygenated, the results would suggest that early in MS there is a diffuse increase in cerebral metabolism, perhaps associated with inflammation, which results in greater oxygen extraction from blood. Patankar et al. show dilated Virchow-Robin spaces (VRS) as representative of white matter abnormalities in patients with subcortical vascular dementia (97). They found more WMH and higher VRS scores in ischemic vascular dementia patients compared with subjects with Alzheimer's disease or healthy controls. Murray et al. show that periventricular lesions had the largest loss of oligodendrocytes and increased levels of microglia (98). They suggest that age related loss of myelin basic protein and small vessel density may lead to vacuolation of WM and accumulation of interstitial fluid.

In the Ganesh and Stahnisch review (4), they note that Schelling suggested mechanical effects leading to “retrograde dilatation of cerebral veins, releasing various immunological phenomena” such as those observed in MS (85). Talbert similarly hypothesized that excessive venous hypertension could stretch the venous wall sufficiently to separate the tight junctions between endothelial cells, allowing colloids and other materials to pass through the exposed porous basement membranes (99). The resulting changes in osmotic pressure could disrupt the internal transport systems of axons and dendrites, leading to their disintegration, triggering the inflammatory processes as in MS. This process might be indistinguishable from those of autoimmune disease forms, which have likewise marked the waxing and waning of experimental autoimmune encephalomyelitis animal models for the study of MS (100).

Dilated veins may be another interesting marker of abnormal flow and MS lesions. As discussed in sections Imaging the Microvasculature Using a USPIO Contrast Agent and Venous Abnormalities Seen With MICRO Imaging and as shown in Figures 1–**3**, the dilated veins and abnormal venous remodeling can by itself serve as a lesion defining marker.

### Endothelial Dysfunction

The dysfunction of the vessel endothelial cells has been proposed to cause alterations of the blood vessel architecture. These changes could lead to both enlargement and narrowing of the vessel lumen, along with vessel stiffening (101). As mentioned earlier, the MS progression and demyelinated lesions have been correlated with increased CBV and vasodilation, which could be caused by the increased endothelial cell proliferation and high levels of vascular endothelial growth factor (VEGF) and VEGF receptors (102) that are associated with the MS population compared to normal controls (103, 104). VEGF production is known to be promoted by several pro-inflammatory cytokines such as interleukin (IL)-1β, IL-1α, and IL-18 (105). In response to increased VEGF, there is an increase in the expression of the endothelial cell adhesion molecules, including the vascular cell adhesion molecules (VCAM) and vascular growth factors; creating a cascade of inflammation and angiogenesis, which promotes the vascular remodeling that is typical to MS. This may result in endothelial junction disorganization, increased iron deposition and immune cell extravasation, which culminates in the loss of neural and glial cells (106, 107). Over time, the chronic MS lesions have been shown to have reduced perfusion due to the reduced axonal activity, lower K+ release in the periaxonal and perivascular space and reduced astrocyte metabolism (104, 108). Hence, in summary, the pro-inflammatory cytokines could cause endothelial dysfunction and an increase in vascular endothelial growth factor and its receptors at early inflammatory stages of the lesion. This can promote the increase in production of vascular adhesion molecules that eventually lead to expediting the loop of inflammation with the increased angiogenesis and/or vascular remodeling. An increase in the production of angiogenic endothelial cells could be due to an endogenous attempt to overcome the chronic hypoperfusion of demyelinating lesions at a late degenerative phase (109, 110) which is linked with the loss of neural and glial cells over time (111).

### Loss of Medullary Vein Density (MVD)

There is also evidence that there is a loss of MVD when comparing relapsing remitting MS (RRMS) to secondary progressive MS (SPMS) to PPMS (95). It has been shown that the MVD decreases supposedly due to poor neuronal function and oxygen extraction and, therefore, leads to reduced levels of deoxyhemoglobin and loss of visibility of the veins in SWI. Another reason could be that the veins are too small to be seen with the current resolution. Zeng et al. also found intra-cerebral venous (ICV) measure of the MVD is highest in CIS and progressively worsens in patients with a longer disease duration (96). They also noted that the periventricular penetrating veins were well-defined in active lesions and ill-defined in non-active lesions. Zhang et al. also present a scoring system for the DMV and find that as the disease progresses the MVD decreases (92). Sinnecker et al. found venous density decreased with increasing lesion count and this loss of medullary veins was already present in CIS to some degree (112).

Although it is not yet clear if this loss of visibility is a marker for continued loss of flow or tissue atrophy, it is yet another marker in the importance of the venous system, especially in the progression of MS.

### Developmental Venous Anomalies (DVAs)

DVAs contain veins that are dilated, twisted and follow a chaotic pattern. They are often referred to as Caput Medusa and sometimes they take on the look of the spokes of a wheel. Here we will focus on evidence of spontaneous vascular remodeling and refer to these as atypical DVAs. This finding may indicate a more serious effect on the surrounding tissue compared to the usual congenital DVAs. Kroll et al. note that devastating venous infarctions after ligation of a DVA confirms that it serves as the only draining route for its corresponding brain segment (113). They also suggest that those DVAs with increased flow (and associated venous congestion likely due to a local venous stenosis) and effects on the surrounding parenchyma should be considered as atypical DVAs (which are more at risk for complications such as hemorrhage or thrombosis). The authors suggest flow may provide a better means for diagnosing an increased risk of associated complications.

Several papers have reported signal intensity abnormalities in and around the tissue in which the DVA is embedded (26, 114) and particularly seen with FLuid Attenuation Inversion Recovery (FLAIR) (115), although Sahin et al. found contrast enhancement and perfusion in the tissue around the DVA in the basal ganglia (116). Umino et al. found 25% of DVAs showed high signal WMH on FLAIR suggesting significant underlying WM disease (117). There was a significant correlation between patient age and the size of WMH abnormalities (117); the age dependence being likely due to time after onset. Linscott et al. showed that deep venous DVAs were more predictive of signal abnormalities (118). Okudera et al. proposed they arose from aplasia, hypoplasia, or occlusion of some part of the medullary venous system or from a pial vein prior to opening to a dural sinus (and these authors also note it could be from chronic venous hypertension caused by anomalous venous drainage) (73). The presence of the bright signals may be from edema, demyelination or gliosis related to abnormal flow from the venous stenosis (117). They ask the key question: “How does a DVA change or grow in time?” Their cross-sectional results suggest that the size increases with age and, hence, they may grow in time. Could the increased resistance to venous outflow lead to leukoaraiosis and collagenous thickening of the venous wall thereby leading to chronic local ischemia and tissue edema? Santucci et al. found non-hemorrhagic signal changes in 12.5% of cases and an association with age (114). Both research teams found one of their patients who met their criteria had MS (114, 117). A very recent study showed nearly 30% of MS patients showed DVAs compared to a control group of headache patients with only 14% of cases (119). More recently using post-contrast T1 weighted imaging and FLAIR, Kruczek et al. confirmed that DVAs are more common in CIS and early-onset MS groups compared to controls, however, prevalence of DVA was not related to other imaging markers, or conversion from CIS to clinically definite MS (120). They found roughly 29% of lesions were DVAs and 21/25 cases had at least one DVA. Nevertheless, the authors note the lack of sensitivity of the sequences they used and recommended the use of other sequences such as SWI (120).

Several groups used perfusion weighted imaging (PWI) and showed many DVAs had increased CBF, CBV, MTT, and Tmax (121–123). Iv et al. postulate that DVAs with intrinsic arterial spin labeling (ASL) signal or signal in draining veins may be associated with arteriovenous shunting (transitional lesions) (123). Kroll et al. used computer tomography (CT) perfusion and found increased CBV, CBF and MTT in the vicinity of the DVA (113). They suggest two types of DVAs: a benign type and one that can lead to tissue damage through ischemia and venous congestion (113). These can be referred to as typical (uncomplicated) and atypical DVAs, respectively. Stenoses, dystrophic calcifications, and cavernous venous malformations are all considered atypical and cavernous malformations (CM) are also considered to be the leading cause of intracerebral hemorrhage. This altered flow pattern seen in PWI may represent venous congestion caused by stenosis of the main draining vein, likely the core etiology in the development of symptomatic DVAs. These atypical DVA may also serve as risk factors for future hemorrhage. Hong et al. suggested that the angioarchitecture of rapidly curving, narrowing and tortuous veins could be risk factors for the formation of cavernous malformations. If two or more of these factors are present, then the risk is much higher. San Millán Ruíz et al. studied the drainage territories of DVAs and their associated parenchymal abnormalities (124). They suggest that outflow obstruction, thickening of venous walls and convergence may lead to the development of venous hypertension in DVA. Stenoses and venous hypertension can lead to demyelination, degenerative alterations of nerve cells, gliosis and leukomalacia around DVAs. Although the medullary veins in the periventricular white matter are ubiquitous, the key finding is that the white matter abnormalities appear to develop along the veins and that they often define the lesion shape as we also see with the micro DVAs (125).

Typical DVAs are usually large venous structural and flow anomalies. Atypical DVAs may be far more serious and in the case of MS, evidence is mounting that they are related to the remodeling alluded to in the above sections and several examples of which are clearly shown in MICRO imaging of MS lesions as discussed in sections Imaging the Microvasculature Using a USPIO Contrast Agent and Venous Abnormalities Seen With MICRO Imaging.

### Vascular Changes as a Pre-Cursor to the Formation of Acute Lesions

Guttman et al. performed an 8 week longitudinal study and found that a stenosis of the vein could be observed on SWI at the time of enhancement on post-gadolinium T1-weighted images, suggesting that vein narrowing may be occurring before BBB disruption (126). There may also be some evidence that intralesional vein stenosis is reversible and consistent with focal hypercellularity in the context of T-cell aggregation during MS plaque formation. In our recent microvascular imaging work, we are seeing local partial inflammation along the outside of the veins that has not yet extended to the entire length of the vein. Finally, another recent study shows that there are changes in the local blood volume in the brain that serve as a pre-cursor to future lesion development before inflammation is seen (127). They provide strong evidence for the role of venous changes pre-plaque development, showing higher venous volumes 1 year before plaque detection and the corresponding NAWM regions (127). Interestingly, they also show that venous volumes are higher even in established plaques and NAWM. Wuerfel et al. assessed around 15 days before MS plaque formation leads to a significant decreased perfusion in the same area. In addition, Holland et al. (48) showed that plaques disappear when brain perfusion is good, and this demonstrates the possibility of myelin repair on behalf of the oligodendrocyte when the oxygen level is adequate (45). It seems that low perfusion is a necessary element for either plaque formationor lesion dissemination.

This recent MRI work presents more direct evidence in MS patients that flow abnormalities occur prior to an inflammatory response. Based on the above material, this vascular effect may well be from abnormal flow in the medullary veins.

### Imaging the Microvasculature Using a USPIO Contrast Agent

We recently introduced a new approach to image vessels as small as 50 to 100 microns in size. This approach makes it possible to study microvascular disease only matched before using cadaver brain studies. We use Ferumoxytol with a dose of 4 mg/kg to increase the susceptibility of the veins and add susceptibility to the arteries so that all vessels are visible with SWI (see Figure 1). The increased susceptibility induces a major signal loss for the small vessels that are less than a voxel in size (128, 129). By using a resolution of 220 microns we can see vessels smaller than 100 microns at 3T, demonstrating the pitchfork (candelabra) behavior of the vessels that matches with the illustrations from the earlier cadaver brain work (Figure 2). USPIO contrast agents have been used before to study MS. These studies showed that more lesions could be found with USPIOs than with gadolinium and postulated that the lesions seen only with USPIO were representative of local macrophage activity otherwise invisible to normal imaging (130–132).

MICRO imaging has been used to visualize the microvascular (predominantly venous) abnormalities in MS lesions. The hypothesis was that using MICRO imaging it will be possible to see the complete venous vascular tree and, hence, be able to study the MS lesions for the presence of vascular abnormalities much better than has ever been done before *in vivo*. MICRO imaging has the potential to study the microvasculature of MS lesions and to follow vascular changes longitudinally over time similar to previous studies (133) to determine which comes first: abnormal vessel structure and flow or inflammation that then affects the vessel wall (129, 134, 135). Or, perhaps both contain some level of guilt and they create a destructive feedback loop exacerbating the disease. The ability to visualize the microvasculature of MS lesions could provide novel information in the development and progression of MS lesions (135).

### Venous Abnormalities Seen With Micro Imaging

Although the presence of Ferumoxytol increases visibility for both arterial and venous vascular network, there is a much higher probability of a vein being present inside the MS lesions as opposed to an artery. Most of the MS plaques are distributed in the WM (136–138) and the arterial blood in the WM is supplied through arterial branches arising from the cerebral arteries, which are roughly 100 μm in size making them very difficult to visualize, especially once they branch into much smaller arterioles and capillaries (72). On the other hand, the medullary veins, subependymal veins and other subcortical WM veins have their primary confluence located in the periventricular region, where the WM lesion occurrence is the highest (138, 139) making them much larger, at ~300 μm in size, than the arterial network in the same region (72). Additionally, the venous blood volume is roughly four times that of the arterial vasculature (140–146), leaving little signal to arise from the arteries. Nevertheless, there is still a need to confirm whether the vascular anomalies have only venous origins. Hence, the pre-contrast SWI data should be used to confirm that these vascular anomalies are part of the venous network.

We have seen a number of key venous vascular abnormalities in our MICRO imaging data even for just the first five patients scanned (see Figure 3) including:

***a) Angiomas:*** Several small/micro-angiomas have been observed in the 5 cases we have done to date. The extent of the angioma predicts the region of inflammation seen in the FLAIR data (Figure 3A). The presence of these small DVAs or venous anomalies is not inconsistent with the fact that it is just those lesions with higher CBV that tend to show as acute lesions (47, 58).
***b) Micro-angiomas:*** We also observed several lesion-centric angiomas-like vessel behavior, where fewer (~3–4 vessels), much smaller (1 voxel-wide visibility) veins show a hint of an irregular, spoke-like vascular pattern that we expect from an angioma (Figure 3B). We termed this category as “micro-angiomas,” which were visible due to the high-resolution post-contrast SWI data.
***c) Engorged/dilated vessels:*** We note that not only is the CVS often present but using MICRO imaging we can see that veins can be locally dilated and it is exactly in this region where FLAIR lesions appear (Figure 3C). The SWI data must be minimum intensity projected (mIP) over a few neighboring slices to increase the visualization along the axis of the vein and to confirm the abrupt changes in its diameter.
***d) Small ovoid lesions:*** Similarly, we observed local WMHs that only partially encapsulate the vein, unlike the usual CVS (Figure 3D). These were smaller in size (<3 mm in length) than the other WMHs and, due to their smaller size, are generally not considered during MS lesion classification (76). These local WMH suggest collagenous thickening of the vessel wall and, as Lumsden notes, these lesions may enlarge and coalesce into a larger plaque over time (147). In fact, an early paper at 7T already showed evidence of small lesions that appeared along the veins but there was no enhancement when gadolinium was used (56).
***e) Perpendicular vessel connections:*** In one subject, a medullary vein perpendicular to the usual venous drainage pathway was present perhaps due to local obstructed flow (again a form of vascular remodeling). Interestingly, these cases were located at the boundary of what appears to be the corona radiata, identified by a reduction in intensity from medial-to-lateral direction in the WM on FLAIR and SWI-FLAIR images (Figure 3E).

These various abnormalities are possible manifestations of poor flow and venous remodeling, all consistent with the previous sections, all perhaps indicative of microvascular effects in the developing MS lesions.

## Summary Of The Putative Timeline Of Events In Ms

**Figure 4:**
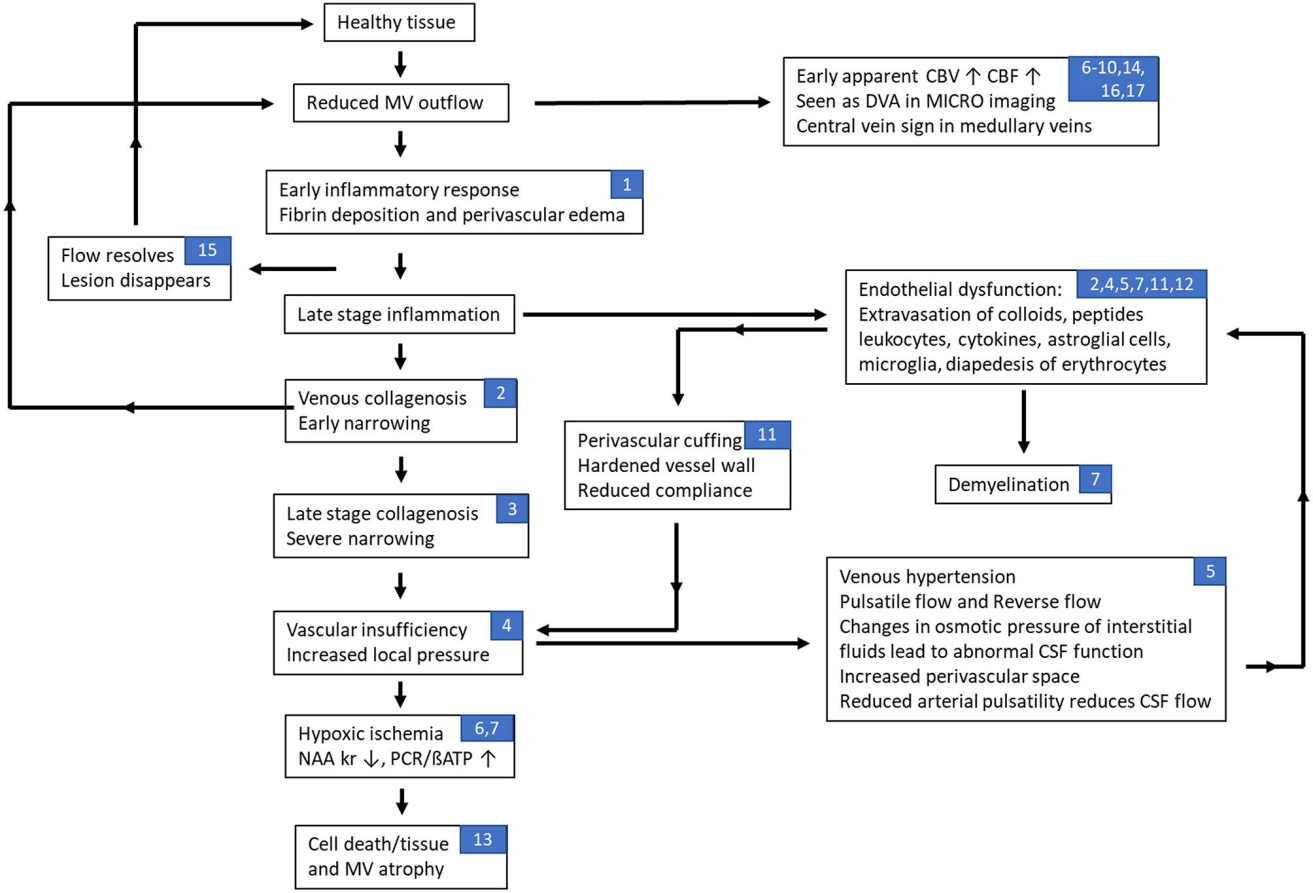
Flow chart of hypothesized effects of abnormal flow in multiple sclerosis related to the neurovascular unit. In this scenario, abnormal medullary vein flow not only initiates the process of vessel wall damage and vasculitis but also can cause remodeling where the brain attempts to overcome the flow abnormalities and, in the process, can create complicated drainage pathways including very small or micro venous angiomas by recruiting anastamotic veins connecting the medullary veins. A recovery of flow may then lead to the resolution (disappearance) of the lesion. Continued slow flow or obstructed flow is likely to exacerbate the conditions leading to blood brain barrier breakdown and worsening of the flow creating a negative feedback loop that eventually leads to cell and tissue death. On the other hand, at early stages of the lesion, the pro-inflammatory cytokines can induce endothelial impairment through increased levels of vascular endothelial growth factor (VEGF), VEGF receptors and endothelial adhesion molecules that lead to a recursive loop that promotes vascular remodeling, angiogenesis and inflammation; especially as the lesion progresses to a late neurodegenerative stage with chronic hypoperfusion. Note that the numbers listed in the blocks refer to the different sections in this paper associated with that stage of the disease process: (1) inflammation and vascular damage; (2) venous collagenosis; (3) role of venous ischemia; (4) cerebral venous infarction; (5) dural sinus flow effects, abnormal CSF flow, and increases in venous pressure; (6) changes in perfusion of MS lesions; (7) fibrin deposition; (8) retinopathic vascular abnormalities; (9) medullary vein flow; (10) central vein sign; (11) evidence of dilated veins; (12) endothelial dysfunction; (13) loss of medullary vein density; (14) developmental venous anomalies; (15) early vascular changes as a marker for new lesions; (16) advanced microvascular MRI using ultra-small superparamagnetic iron oxides (USPIO); and (17) imaging indications of venous abnormalities.

The various materials introduced above include inflammation, vasculitis, abnormal flow, fibrin and collagen deposition leading to further reduced flow and perivascular medullary vein involvement. Recent imaging evidence provides the foundation stones for building a potential timeline for events that may represent some aspects of the pathophysiology of multiple sclerosis. Although the key source of MS is unknown, we make the assumption that it is, in fact, initially related to abnormal flow and the cascade of events that would follow including vasculitis, inflammation, demyelination, ischemia and eventual tissue death. Based on all these works, we present our interpretation of the timeline of events in Figure 4. In this scenario, abnormal medullary vein flow not only initiates the process of vessel wall damage and vasculitis but also can cause vascular remodeling where the brain attempts to overcome the flow abnormalities and, in the process, can create complicated drainage pathways including very small or micro venous angiomas by recruiting anastamotic veins connecting the medullary veins. Sosa et al. show that the abnormal flow can initiate an endothelial response which in turn can cascade into further vascular damage and flow obstruction eventually leading to an hypoxic ischemic situation which in the case of chronic inflammation finally leads to the destruction of the tissue (148). They also note that the anterior and posterior horns are particularly vulnerable because they are at the watershed zone of the anterior and middle cerebral arteries (148). Using an animal model, Desai et al. note that lesions tended to form specifically in areas of hypoxia and that by reducing the level of hypoxia lesions were less likely to form (149). These observations are complementary to the discussion in Caprio et al. in their review of cardiovascular risk factors related to MS (3). They consider the roles of smoking, hypertension, diabetes, vitamin D, etc. They also note that there are elevated levels of endothelin-1, a potent vasoconstrictor, in the plasma of MS patients. They conclude that endothelial cell adhesion molecules, pro-inflammatory cytokines, vascular growth factors and other molecules could contribute to vascular remodeling that is typical of MS, which can be exacerbated during chronic hypoperfusion in later stages of the lesion progression.

## Future Possibilities And Other Considerations

Subjects diagnosed with radiologically isolated syndrome (RIS) can be described as asymptomatic individuals with incidental radiologic abnormalities suggestive of MS and we have focused on the studies that cover the pathogenesis for the subjects that have already clinical signs related to the MS. Nevertheless, there have been studies that show that in subjects with RIS, around one-third of all subjects developed neurological symptoms within 5 years (150). Another recent study examined the occurrence of CVS in RIS subjects and found the number of CVS in RIS much higher (75%) than the 40% rule in the MS subjects reflecting a higher rate of perivenous inflammatory demyelination (151). This reduction in CVS from 75% in RIS to 40–50% in MS could suggest that, for the subjects that do evolve to symptomatic MS, the venous collagenosis developed over time due to the surrounding inflammation and this led to venous wall thickening, luminal narrowing and vessel occlusion. However, their subject size was small (*n* = 20) and the prospective follow-up study has not been published yet to confirm how many showed neurological symptoms similar to MS. We would need a larger population to definitively conclude that venous process.

A key question to resolve in the future with a longitudinal study would be: “Are the small lesions that appear around the normal appearing vessels representative of the development of future lesions?” With the help of longitudinal MICRO data, we could further elucidate whether the abnormal vessel behavior, within or surrounding the lesion, act as a potential source initiating the inflammatory response. Finally, the automatic separation of venous and arterial components of the microvasculature for MICRO imaging is an important challenge to address. This will then allow us to separate the effects of arterial or venous network on the demyelination over time. One could potentially achieve this by acquiring SWI at multiple timepoints with increasing Ferumoxytol concentration levels to elucidate the difference in temporal signal changes for arterial and venous blood pools.

## Conclusion

All this evidence of venous vascular abnormalities in MS leads to a critical hypothesis as to one potential cause of MS: “*Local disrupted venous flow leads to remodeling of the medullary veins followed by a breakdown of the endothelium with the subsequent escape of glial cells, cytokines, etc. that in turn lead to the autoimmune demyelinating process and subsequent tissue death and atrophy.”* There are several key features in MS that can be understood in the context of this review. Whether or not venous flow disruption occurs first, the pathological course of MS is consistent with the continual feedback loop of constantly reducing blood flow over time. This is consistent with all findings in the literature. There are multiple mechanisms by which demyelination could occur at the cellular level but the source likely remains the extravasation of various cells and how they might precipitate demyelination. Abnormal flow is also consistent with generating an endothelial response in the form of inflammation first. This then leads again to the conventional wisdom on how the endothelium responds to inflammation. However, in the scenario outlined here, we show the effects of continued flow reduction because of processes such as fibrin deposition and collagenosis. This can then create a feedback loop as shown in Figure 4 where vascular insufficiency leads to venous hypertension and other flow effects that then lead to a breakdown of the BBB. Further, the return to normal flow may, in fact, predict why lesions can recover as the usual nutrients and conditions required for endothelial health return to normal as well. In summary, this comprehensive overview of vascular effects in MS should open the door to study abnormal flow and its temporal relationship to the development of MS lesions using a variety of *in vivo* imaging methods. We encourage further research to study the microvasculature in MS as it relates to lesion formation and the development of chronic lesions.

## Author Contributions

EH wrote the first draft of the manuscript. EH, YG, SS, SB, and PZ prepared, critically discussed the review, and edited the manuscript. All authors contributed to the article and approved the submitted version.

## Conflict of Interest

EH and SS are employed by SpinTech, Inc. and MR Innovations, Inc. The remaining authors declare that the research was conducted in the absence of any commercial or financial relationships that could be construed as a potential conflict of interest.
